# Correlates of Glycemic Control Among Patients With Type 2 Diabetes in Eastern Ethiopia: A Hospital-Based Cross-Sectional Study

**DOI:** 10.3389/fendo.2022.939804

**Published:** 2022-07-22

**Authors:** Shiferaw Letta, Fekadu Aga, Tesfaye Assebe Yadeta, Biftu Geda, Yadeta Dessie

**Affiliations:** ^1^ School of Nursing and Midwifery, College of Health and Medical Sciences, Haramaya University, Harar, Ethiopia; ^2^ School of Nursing and Midwifery, College of Health Sciences, Addis Ababa University, Addis Ababa, Ethiopia; ^3^ Department of Nursing, College of Health Sciences, Madda Walabu University, Shashamene, Ethiopia; ^4^ School of Public Health, College of Health and Medical Sciences, Haramaya University, Harar, Ethiopia

**Keywords:** glycemic control, type 2 diabetes, total cholesterol, triglycerides, Harar, Ethiopia

## Abstract

**Introduction:**

Even though optimal blood glucose control reduces the risk of diabetes-related complications, many patients with type 2 diabetes (T2D) fail to achieve it for a variety of reasons. In the study area, there was a paucity of evidence regarding correlates of glycemic control. Therefore, this study aimed to find out the correlates of glycemic control among patients with T2D in Eastern Ethiopia.

**Methods:**

A cross-sectional study was conducted among 879 adult patients with T2D on follow-up at two public hospitals in Harar. Data were collected through interviews, physical measurements, and record reviews. The level of glycemic control was determined from three consecutive fasting plasma glucose (FPG) measurements. A mean value of FPG measurements falling in the normal range (80–130 mg/dl) was considered as optimal glycemic control; otherwise, a mean FPG level that is below or above the normal range (<80 mg/dl or >130 mg/dl) was defined as suboptimal glycemic control. Descriptive statistics were used to summarize the data, while a linear regression model was used to find out the correlates of glycemic control. A beta coefficient and a 95% CI reported associations. The statistical significance was declared at a p-value ≤0.05.

**Results:**

The mean age of the patients with T2D was 52.7 ( ± 13.3) years. The mean FPG level was 172 ± 56 mg/dl. Suboptimal glycemic control was found in 76% (95% CI: 73.41, 79.04) of patients with T2D. In a multivariable linear regression, khat chewing (β = 6.12; 95% CI: 1.55, 8.69), triglycerides (β = 0.56; 95% CI: 0.41.48, 0.65), comorbidity (β = 5.29; 95% CI: 1.39, 9.13), and poor level of self-care practices (β = 5.43; 95% CI: 1.41, 6.46) showed a significant correlation with glycemic control.

**Conclusions:**

This study found that about three-fourths of patients with T2D had suboptimal glycemic control. Khat chewing, comorbidity, and poor level of self-care practices were independently correlated with glycemic control. Thus, suppressing glycemic levels through appropriate treatment and strict diabetes self-care practices including avoidance of Khat chewing is a useful approach to attaining glycemic target that subsequently reduces cardiovascular risks.

## Introduction

Diabetes is one of the fastest-growing global health emergencies of the 21st century that has reached alarming levels (1). Type 2 diabetes (T2D) is a progressive illness associated with decreasing insulin secretion over time (2), and poorly controlled diabetes leads to multiple organ damage that can increase the overall risk of premature death (3).

Evidence shows that optimal glycemic control is a goal for diabetes management. The general target of glucose control is glycated hemoglobin (HbA1c) ≤7% for non-pregnant adults, and a less stringent HbA1C goal of 8% (64 mmol/mol) is optional for patients with a risk of severe hypoglycemia and advanced microvascular or macrovascular complications (4). However, HbA1c is expensive and unavailable in many places. Therefore, a fasting plasma glucose (FPG) level of 80–130 mg/dl without caloric intake for at least 8 h is one of the standard diagnostic criteria (5).

Growing evidence showed that optimal blood glucose control prevents acute and chronic complications, whereas a sustained hyperglycemic condition results in a severe diabetic condition by damaging the pancreatic β-cell and inducing insulin resistance (6, 7). Though a strong agreement, most diabetic patients do not attain their diabetic goals (8, 9). These problems are prominent in resource-limited countries including Ethiopia where access to diabetes health services and standard laboratory tests were inadequate (10).

The government of Ethiopia has made several efforts through policy formulation, designing strategies to identify risk factors, and developing management protocol for diabetes (7, 11); so far, two-thirds of patients fail to attain optimal glycemic control due to complex reasons (9). The most recurrently reported factors influencing glycemic control were younger age, male sex, marital status, rural residence, longer duration of diabetes, poor level of medication adherence, and self-monitoring blood glucose (9, 12–16). Specifically, studies indicated a higher prevalence of T2D and metabolic syndromes along with numerous barriers to self-care practices in the study area (17–19). Khat chewing habit has also been popular (20). However, evidence about correlates of glycemic control was scarce. Therefore, this study aimed to find out the correlates of glycemic control among patients with T2D in Eastern Ethiopia. This knowledge would provide valuable information for healthcare providers, local planners, and implementers to plan appropriate interventions that improve patients’ glycemia and lipid levels to reduce the extent of target organ damage.

## Materials and Methods

### Study Setting and Design

A cross-sectional study was conducted among patients with T2D at public health hospitals in Harar between December 1, 2020, to March 30, 2021. Harar city is found in the eastern part of the country, 526 km away from Addis Ababa. Hiwot Fana Specialized Comprehensive Hospital and Jugal General Hospital are two public hospitals that offer comprehensive health services for the entire community of eastern Ethiopia. The hospitals deliver services in medical, surgical, pediatric, obstetrics, and gynecology wards; intensive care units (ICUs); outpatient departments (OPDs); and radiology, pathology, laboratory, and pharmacy departments. Nursing services are also provided in all departments of the hospital with a team of allied disciplines in delivering patient care that aims at satisfying the nursing needs of the patients. Moreover, the hospitals have trained health and medical science students. In these hospitals, more than 1,985 patients with T2D were attending their follow-up care.

### Population

Adult patients with a diagnosis of T2D who had diabetes follow-up at Hiwot Fana Specialized Comprehensive Hospital and Jugal Hospital were involved in the study. The patients with T2D who had at least three consecutive FPG measurements were randomly selected. However, two patients with T2D with severe illness and hearing impairment were excluded from the study, as they might not respond to our questions accurately and were unable to give valid consent.

### Sample Size and Sampling Strategy

The estimated sample size was determined using a single and double population proportion formula using Epi-info version 7.1 software, where n is the sample size, Zα/_2_ = 1.96, d = 0.05 is the margin of error, P = 64.7% for the level of suboptimal glycemic control (12). Accordingly, a total of 891 patients with T2D were included in this study (598 = Jugal Hospital and 293 = Hiwot Fana Specialized Comprehensive Hospital). Then, the study participants were recruited randomly every two, while the first participant was selected by lottery method.

### Data Collection and Measurements

Data were collected by interviewing eligible participants using a pretested semistructured questionnaire after reviewing various literature. The questionnaires consisted of demographic information and clinical characteristics diabetes duration, current regimen, comorbidity, complications, and biochemical data such as FPG, total cholesterol, and triglyceride levels. Summary diabetes self-care activity (SDSCA) has physical activities, dietary plan, medication adherence, blood glucose monitoring, foot care, smoking behaviors, and alcohol use (21). A wealth score was also computed using principal component analysis (PCA) from 23 variables that included household assets, farmland, and animals.

The blood glucose levels were determined by collecting the three most recent FPG measurements in the last three consecutive visits and calculating the mean values. The measurement of blood glucose was conventionally classified into two categories (controlled vs. uncontrolled or optimal vs. suboptimal). Based on the American Diabetic Association (ADA) standards of medical care, optimal glycemic control is an average FPG level of 80–130 mg/dl and suboptimal glycemic control refers to an average FPG level of <80 mg/dl and >130 mg/dl (4).

Blood pressure (BP) measurements were carried out using a digital automated BP monitor (Model UA-767F/UA-767FAC, manufactured by A&D Company, Limited, Japan) with patients sitting after resting for at least 15 min. BP measurement was made on the left arm with the elbow supported and the palm facing upward. Three consecutive measurements of BP were taken in a 3-min interval, and then the mean of the second and third readings was used for analysis (22). BP measurement ≥140/90 mmHg indicates raised BP or hypertension (23).

Anthropometric data [height, body weight, hip circumference (HC), and waist circumference (WC)] were collected by using standard procedures and calibrated instruments at the end of the interview. Height was measured using a stadiometer with removed footwear and hair gear and the patient’s face away from the wall, looking straight ahead with his or her heels together and the back as straight as possible. The head, shoulders, buttocks, and heels should be in contact with the vertical surface, and the height measurement was recorded to the nearest 0.1 cm. Body weight was measured barefooted and wearing light clothes using a Seca digital body weight scale made in Japan and measuring to the nearest 0.1 kg. Body mass index (BMI) was calculated as the patient’s weight in kilograms divided by height in square meters (kg/m^2^). BMI was categorized into four: underweight (<18.5 kg/m^2^), normal (18.5–24.9 kg/m^2^), overweight (25.0–29.9 kg/m^2^), and obese (≥30 kg/m^2^) (22).

WC was measured in centimeters using a fixed tension tape at the midpoint of the line between the lower margin of the last palpable (12th) rib and the top of the iliac crest (hip bone) over light clothing without compressing the skin. The measurement was taken at the end of an expiration with the arms relaxed at the sides, and the measurement was recorded to the nearest 0.1 cm. The WC measurements were classified based on cutoffs recommended by the WHO into three health risk categories: low risk (men, WC = ≤93.9 cm; women, WC = ≤79.9 cm); increased risk (men, WC = 94.0–101.9 cm; women, WC = 80.0–87.9 cm); and high risk (men, WC = ≥102.0 cm; women, WC = ≥88.0 cm) (22).

HC was taken around the maximum circumference of the buttocks while the patients stand with their feet together with weight evenly distributed over both feet and holding their arms relaxed at the sides. The HC was measured using an inelastic measuring tape and recorded to the nearest 0.1 cm. Then, the waist-to-hip ratio (WHR) was calculated by dividing WC by HC in centimeters. The cutoff point used for WHR was ≤0.9 for the men and ≤0.85 for the women (22).

### Data Quality Control

The data were collected using a standardized questionnaire written in English and translated into local languages. After that, it was retranslated into English by another expert to confirm that the instruments were consistent. Before the actual fieldwork, data collectors and supervisors were trained to ensure reliable and accurate data collection. The research instrument was also pretested in 50 patients with T2D who visited Dilchora Hospital for diabetic care, which is found about 52 km away from the study area. Moreover, the supervisors and principal investigator double-checked the data for completeness daily throughout the data collection period.

### Statistical Analysis

The collected data were cleaned, coded, and entered to Epidata software version 3.1 and then exported to Stata version 14.0 for analysis. Descriptive statistics such as mean and standard deviation were used to report data of continuous variables, whereas frequencies and percentages were used to express categorical variables. A variable with p < 0.20 on a bivariate linear regression analysis was entered into a multivariate linear regression analysis model to identify the variable independently correlated with glycemic control. The data were summarized using beta coefficient with a corresponding 95% confidence interval. A variable with p ≤ 0.05 was statistically significant. The multicollinearity test was carried out to see the linear correlation among independent variables. The variance inflation factor and correlation coefficient did not prove the existence of multicollinearity. Model fitness was checked using the Hosmer–Lemeshow goodness-of-fit test.

## Results

### Demographic Characteristics

A total of 879 patients with T2D were included in the study, yielding a response rate of 99%. The female patients accounted for 56.1%. The mean age was 52.7 ± 13.3 years. Four hundred forty-seven (50.9%) were above the age of 55 years. Three hundred fifty-two patients (40.1%) had no formal education. Six hundred eighty-four (77.8%) of them were urban dwellers. Three hundred forty-five patients (39.4%) were in the medium wealth quantile category, while two-thirds (65.6%) of them had no health insurance coverage. The mean FPG level indicated persistently high glycemic levels across all demographic characteristics of the patients with T2D (**Table 1**).

**Table 1:** Demographic characteristics by glycemic levels of patients with T2D in Eastern Ethiopia, 2020/2021 (n = 879).

Variables	Category	n (%)	FPG level
			Mean (SD), mg/dl
Sex			
	Men	386 (43.9)	169.6 (58.7)
	Women	493 (56.1)	173.3 (53.4)
Age, mean ± SD (years)		52.7 ± 13.3	
	18–34	83 (9.4)	168.3 (66.7)
	35–44	128 (14.6)	182.7 (56.0)
	45–54	221 (25.1)	174.6 (53.9)
	55+	447 (50.9)	167.7 (54.1)
Educational level			
	No formal education	352 (40.0)	172.7 (56.1)
	Primary (Grades 1–8)	216 (24.6)	177.1 (53.2)
	Secondary (Grades 9–12)	171 (19.5)	163.6 (58.7)
	Tertiary (12^+^)	140 (15.9)	163.6 (57.2)
Marital status			
	Never married	72 (8.2)	166.1 (55.3)
	Married	714 (81.2)	171.3 (55.3)
	Divorcee and widowed	96 (10.6)	176.7 (56.8)
Occupation			
	Paid employee	209 (23.7)	169.7 (55.6)
	Merchant	74 (8.4)	171.7 (56.5)
	Farmer and daily laborer	203 (23.1)	178.1 (59.4)
	Housewife	295 (33.6)	181.7 (52.2)
	Others*	98 (11.2)	170.4 (53.4)
Residence			
	Urban	684 (77.8)	171.0 (54.1)
	Rural	195 (22.2)	173.9 (61.7)
Wealth index quantile			
	Low	296 (33.7)	172.7 (57.3)
	Medium	345 (39.4)	167.5 (56.3)
	High	237 (26.9)	176.6 (52.0)
Health insurance			
	No	577 (65.6)	170.1 (56.1)
	Yes	302 (34.3)	174.7 (55.3)

*Others: non-employee, students, and retired.

In this study, 76% (95% CI: 73.4, 79.0) of T2D patients had suboptimal glycemic control (<80 mg/dl or >130 mg/dl). The average FPG level was 172 ± 56 mg/dl (95% CI: 167.9, 175.4). Female T2D patients showed slightly worse glycemic control than did male patients.

### Clinical Characteristics

The mean glycemic control based on FPG level was 172.5 ± 56.2 mg/dl among patients with T2D who had less than 5 years of disease duration. The mean FPG level of patients with T2D who had no comorbidity was 173.9 ± 55.9 mg/dl. Although 300 (43.2%) patients with T2D were being treated with a combination of oral glycemic control agents (OGAs), their mean FPG level was 171.9 ± 55.7 mg/dl. The mean FPG level among patients with T2D who had no history of admission was 172.8 ± 56.5 mg/dl. More than half (58.8%) of patients with T2D with normal total cholesterol levels (<200 mg/dl) had a mean FPG level that was closer to normal (138.9 ± 33.9 mg/dl). Moreover, 315 (35.8%) patients with T2D had high to very high levels of triglycerides, and the corresponding mean FPG level was high (227.1 ± 40.9 mg/dl), and 65.7% of patients were found to have poor self-care practices with a mean FPG level of 173.5 ± 57.5 mg/dl (**Table 2**).

**Table 2:** Clinical characteristics by mean FPG level of the patients with T2D in Eastern Ethiopia, 2020/2021 (n = 879).

Variables	Category	n (%)	FPG level
			Mean (SD), mg/dl
DM duration, years			
	<5 years	680 (77.4)	172.5 (56.2)
	>5 years	199 (22.6)	168.9 (54.7)
Co-morbidity			
	No	656 (74.1)	173.9 (55.9)
	Yes	223 (25.4)	165.2 (55.5)
Hypertension			
	No	739 (84.1)	172.5 (57.4)
	Yes	140 (15.9)	169.2 (55.2)
Current regimen			
	Insulin monotherapy	217 (24.7)	171.5 (57.4)
	Insulin with OGAs*	133 (15.1)	166.4 (52.6)
	OGA monotherapy	149 (16.9)	175.8 (54.8)
	Combination of OGAs	380 (43.2)	171.9 (55.7)
Glucometer			
	No	699 (79.5)	172.8 (56.1)
	Yes	180 (20.5)	167.4 (54.6)
Admission history			
	No	812 (92.4)	172.8 (56.5)
	Yes	67 (7.6)	157.3 (45.2)
Total cholesterol			
	Normal (<200 mg/dl)	517 (58.8)	138.9 (33.9)
	Borderline (200–239 mg/dl)	96 (10.9)	203.7 (25.4)
	High (≥240 mg/dl)	266 (30.3)	223.8 (51.8)
Triglycerides			
	Normal (150–199 mg/dl)	304 (36.9)	122.8 (25.9)
	Borderline (150–199 mg/dl)	240 (27.3)	164.8 (31.7)
	High and very high (>200 mg/dl)	315 (35.8)	227.1 (40.9)
DM education			
	Not attended	616 (70.1)	171.7 (55.1)
	Attended	263 (29.9)	171.5 (57.4)
Diabetes self-care			
	Poor	531 (65.7)	173.5 (57.5)
	Good	277 (34.3)	170.0 (53.2)

*OGAs, oral glycemic control agents.

### Anthropometric Measurements and Biochemical Tests

The mean ( ± SD) of BMIs of male and female participants were 25.0 ± 3.7 and 27.1 ± 5.5, indicating overweight, respectively. Likewise, the mean FPG, total cholesterol, and triglyceride levels had no significant differences in both men and women (**Table 3**).

**Table 3:** Anthropometric indices and biochemical tests by sex of patients with T2D in Eastern Ethiopia, 2020/2021 (n = 879).

	Men (n = 493)		Women (n = 386)	
Variable	Mean (SD)	SE	Mean (SD)	SE
Age (years)	53.2 (14.1)	0.7	52.4 (12.6)	0.6
WC (cm)	94.6 (66.1)	3.4	94.0 (43.5)	1.9
HC (cm)	92.1 (14.1)	0.7	98.4 (43.4)	1.5
WHR	0.9 (0.1)	0.1	0.9 (0.1)	0.1
BMI (kg/m^2^)	25.0 (3.7)	0.2	27.1 (5.5)	0.3
SBP (mmHg)	127.9 (16.5)	0.8	125.5 (17.2)	0.8
DBP (mmHg)	82.1 (10.0)	0.5	80.2 (10.4)	0.5
Total cholesterol (mg/dl)	196.7 (70.0)	3.1	203.3 (73.3)	3.7
Triglycerides (mg/dl)	191.4 (71.5)	3.7	189.8 (70.2)	3.1

WC, waist circumference; HC, hip circumference; WHR, waist-to-hip ratio; BMI, body mass index; SBP, systolic blood pressure; DBP, diastolic blood pressure.

### Correlates of Glycemic Control in Patients With Type 2 Diabetes

In bivariate analysis, participants’ age, educational level, health insurance, diabetes complication, admission history, attendance of religious fasting, total cholesterol, triglycerides, khat chewing, sugary drinks used during khat chewing, and self-care practices were statistically significant. After adjusting for potential confounding factors, total cholesterol, triglycerides, diabetes complications, chewing khat, and self-care practices showed a statistically significant association with glycemic control.

Keeping constant all variables in the model, an increase in the level of triglycerides by 1 unit increased the FPG level of patients with T2D by 0.51 mg/dl (β = 0.51; 95% CI: 0.48, 0.54). Khat chewing practices in patients with T2D indicated an increase in FPG level by 6.12 mg/dl (β = 6.12; 95% CI: 1.55, 8.69). Having comorbid conditions increased the FPG level of patients with T2D by 5.29 mg/dl (β = 5.29; 95% CI: 1.39, 9.13). In addition, poor levels of diabetes self-care practices increased the patients’ FPG level by 5.43 mg/dl (β = 5.43; 95% CI: 1.41, 6.46), while all variables in the model were kept constant (**Table 4**).

**Table 4:** Correlates of glycemic control among patients with T2D in Eastern Ethiopia, 2020/2021 (n = 879).

Variable		Glycemic control				
		Unadjusted		Adjusted		
	β coef.	95% Conf. Interval		β coef.	95% Conf. Interval	
Age	-0.28	-0.56	-0.01	-0.014	-0.72	0.69
Educational level	-2.82	-6.17	0.52	1.89	-9.26	3.05
Health insurance	10.49	0.72	20.27	-5.55	-10.39	1.28
Admission history	15.56	1.16	29.53	5.23	0.59	6.53
Attendance of fasting	-8.48	-17.78	0.81	6.82	-9.75	9.38
Triglycerides	0.61	0.58	0.64	0.51	0. 48	0.54*
Presence of comorbidity	17.99	9.31	26.67	5.29	1.39	9.19*
Advised on physical exercise	-11.79	-23.91	0.32	-12.78	-22.56	6.99
Poor self-care practices	3.49	1.65	4.67	5.43	1.41	6.46*
Khat chewing	10.54	1.87	19.21	6.12	1.55	8.69**
Sugary drinks while khat chewing	13.09	1.65	24.54	4.69	5.00	7.62

**p < 0.001, *p < 0.05.

## Discussion

This study explored correlates of glycemic control among patients with T2D in Eastern Ethiopia. Accordingly, 76% of patients with T2D (95% CI: 73.4, 79.0) had suboptimal glycemic control. The mean FPG level was 172 ± 56 mg/dl (95% CI: 167.9, 175.4), indicating uncontrolled blood glucose.

The findings of this study indicated that three-fourths of patients with T2D did not achieve the recommended glycemic target (FPG level of 80–130 mg/dl). This informed that most of the patients are at higher risk of diabetes complications (24, 25). The risk of premature death is usually higher in people with comorbidity (26). Its terrible economic loss affects the patients and their families and the national economy through medical costs and loss of work (3).

The finding was consistent with those of previous studies conducted in Saudi Arabia and India (15, 27, 28). The reasons for similarities might be that these studies shared common characteristics: there was a higher proportion of women who naturally have higher adipose tissue mass, free fatty acids, and intramyocellular lipid content that could promote insulin resistance and affect glycemic control (29). Most of the study participants were urban residents who had higher indices of overweight and obesity that potentially contribute to insulin resistance, reduce the uptake of glucose, and result in suboptimal glycemic control. Although regular physical exercise has a crucial role in increasing insulin sensitivity and improving glycemic management (30), physical inactivity (<150 min/week) was rampant. In addition, participants in Saudi Arabia were less educated, while more than three-fourths of patients with T2D had no formal education, which might limit their ability to understand the nature of the disease and access to adequate health care services.

However, the level of glycemic control in the current study was slightly lower than that of Bangladesh (31), Pakistan (32), and Addis Ababa, Ethiopia (33). Compared to Bangladesh and Pakistan studies, the proportion of comorbidities, overweight, and obesity that influence optimal glycemic control was lower. Moreover, the use of combination therapy was higher in the current study, which may influence glycemic control to some extent in contrast to single therapy. There was also a relative increment of the suboptimal level of glycemia in this study when compared with that of previous studies conducted in Mettu, Jimma, and Debre Tabor public hospitals of Ethiopia (34–36). The variation could be due to using a larger sample size, and the use of OGAs was the predominant regimen in the current study.

The current study also found that chewing khat, triglycerides, having comorbidity, and a poor level of self-care practices were independent correlates of glycemic control. Khat chewing showed a strong association with glycemic control. This result was supported by studies conducted in Sana’a and Hodeidah City, Yemen, that the patients who chewed khat demonstrated a higher mean HbA1c than non-khat chewer patients with T2D (37, 38). Another study in Malaysia disclosed that khat (cathinone) significantly increased the FPG level and decreased the insulin level of diabetic rats (39). This could be because most khat chewers used sugary drinks/beverages while chewing (40). Khat also has a cytotoxic effect on pancreatic cells and insulin synthesis, affecting regulation of blood glucose (41). However, there were contradictory findings about its effect on glycemic control (40, 42). Therefore, another study is suggested to determine the actual effects of khat on the blood glucose level.

The triglyceride level was also strongly correlated with glycemic control. This finding was consistent with studies conducted in Mainland and Zhejiang, China, and Nepal (43–45). Similarly, a study in Bosnia and Herzegovina showed a positive correlation between glycemic control and triglyceride level (46). In general, the lipid abnormality in patients with T2D might be explained based on insulin resistance that can occur due to genetic defects, ectopic lipid accumulation, physical inactivity, obesity, and inflammation (47–49). Insulin resistance reduces glycogen synthesis and protein catabolism in skeletal muscles while inhibiting lipoprotein lipase activity in adipocytes, resulting in a higher release of free fatty acids and inflammatory cytokines (50, 51). Moreover, insulin resistance impairs glucose output and fatty acid metabolism, increasing the triglyceride content from the liver (50, 52) and resulting in hypertriglyceridemia and increasing the synthesis of Very Low-Density Lipoprotein (VLDL), which increases cardiovascular risks by causing atherosclerosis (53).

In the current study, a poor level of self-care practices showed a correlation with glycemic control. This finding was comparable to that of a study done in Riyadh, Saudi Arabia, where a poor level of diabetes self-care behavior was associated with suboptimal glycemic control (54), which increases the risk of morbidity and mortality in diabetes patients (55). On the other hand, good self-care practices optimize glycemic control (32), reduce the risk of diabetes complications (56), and improve patients’ quality of life (57). The HbA1c level can be reduced up to 1% through self-care practices (58, 59).

Comorbidity appeared as a correlate of glycemic control in this study. This finding was congruent with those of previous studies conducted in Ethiopia (60) and Croatia that reported the effect of comorbidities on the level of HbA1c (61). This might be because patients with a comorbid condition have less energy to have physical exercise, which likely lowers the blood glucose level (62). Moreover, diabetic patients who had comorbidities received more drugs (63). The use of multiple medications might increase the chance of drug–drug interactions (64) and food–drug interactions (65), decreasing their medication adherence (66) and leading to suboptimal glycemic control (67).

### Strengths and Limitations of the Study

The average of the three most recent FPG measurements was used to determine glycemic levels, which is more reliable than a single measurement. On top of that, an adequate sample size was used for meaningful results/conclusions. However, our study had some limitations. Self-report and social desirability biases might potentially influence the results of the study. In addition, the retrospective nature of chart review was a major limitation.

## Conclusions

The findings suggested that considerable numbers of patients with T2D had suboptimal glycemic control usually linked to diabetes-associated complications. Khat chewing, triglycerides, comorbidity, and a poor level of diabetes self-care practices were independently correlated with glycemic control. Regular monitoring, suppressing triglyceride levels through strict self-care practices, and appropriate treatment can benefit the patients to attain optimal glycemic control that subsequently reduces diabetes-related complications. Diabetes self-management education that focuses on the avoidance of khat chewing needs to be instituted. Furthermore, a controlled trial study that generates robust evidence is recommended to elucidate the effect of khat chewing on the blood glucose level.

## Data Availability Statement

The raw data supporting the conclusions of this article will be made available by the authors, without undue reservation.

## Ethics Statement

The studies involving human participants were reviewed and approved by The study was reviewed and approved by the Institutional Health Research and Ethical Review Committee (IHRERC) of the College of Health and Medical Sciences, Haramaya University with the reference number (IHRERC/2017/2020). In addition, an official letter of cooperation was obtained from the college to get permission from the hospital administrators and diabetes units. The patients/participants provided their written informed consent to participate in this study.

## Author Contributions

All authors had a significant contribution to the conception, study design, execution, analysis, and interpretation of the data; took part in drafting, critically reviewing the article; approved the final version for publication, and agree to be accountable for all aspects of the work.

## Funding

This research was funded by Haramaya University, Ethiopia. The funder had no role in designing and data collection, analysis, writing, and submission of the manuscript for publication.

## Conflict of Interest

The authors declare that the research was conducted in the absence of any commercial or financial relationships that could be construed as a potential conflict of interest.

## Publisher’s Note

All claims expressed in this article are solely those of the authors and do not necessarily represent those of their affiliated organizations, or those of the publisher, the editors and the reviewers. Any product that may be evaluated in this article, or claim that may be made by its manufacturer, is not guaranteed or endorsed by the publisher.
